# Dasatinib synergizes with doxorubicin to block growth, migration, and invasion of breast cancer cells

**DOI:** 10.1038/sj.bjc.6605101

**Published:** 2009-06-09

**Authors:** C S Pichot, S M Hartig, L Xia, C Arvanitis, D Monisvais, F Y Lee, J A Frost, S J Corey

**Affiliations:** 1 Department of Integrative Biology and Pharmacology, University of Texas Health Science Center, Houston, Texas, USA; 2 Department of Cell Biology, Baylor College of Medicine, Houston, Texas, USA; 3 Department of Surgical Oncology, University of Texas MD Anderson Cancer Center, Houston, Texas, USA; 4 Departments of Pediatrics and Cellular and Molecular Biology, Children's Memorial Hospital and Robert H. Lurie Comprehensive Cancer Center, Northwestern University, Chicago, Illinois, USA; 5 Bristol-Myers Squibb Pharmaceutical Research Institute, Princeton, New Jersey, USA

**Keywords:** dasatinib, Src, invasion, invadopodia, breast cancer

## Abstract

**Background:**

Src family kinases control multiple cancer cell properties including cell cycle progression, survival, and metastasis. Recent studies suggest that the Src inhibitor dasatinib blocks these critical cancer cell functions.

**Methods:**

Because the molecular mechanism of action of dasatinib in breast cancers has not been investigated, we evaluated the effects of dasatinib as a single agent and in combination with the commonly used chemotherapeutic doxorubicin, on the proliferation, viability, and invasive capacity of breast cancer cells lines earlier categorised as dasatinib-sensitive (MDA-MB-231) and moderately resistant (MCF7 and T47D). We also tested the effects of these drugs on the actin cytoskeleton and associated signalling pathways.

**Results:**

The cell lines tested varied widely in sensitivity to growth inhibition (IC_50_=0.16–12.3 *μ*M), despite comparable Src kinase inhibition by dasatinib (IC_50_=17–37 nM). In the most sensitive cell line, MDA-MB-231, dasatinib treatment induced significant G_1_ accumulation with little apoptosis, disrupted cellular morphology, blocked migration, inhibited invasion through Matrigel (*P*<0.01), and blocked the formation of invadopodia (*P*<0.001). Importantly, combination treatment with doxorubicin resulted in synergistic growth inhibition in all cell lines and blocked the migration and invasion of the highly metastatic, triple-negative MDA-MB-231 cell line.

**Conclusion:**

The observed synergy between dasatinib and doxorubicin warrants the re-evaluation of dasatinib as an effective agent in multi-drug regimens for the treatment of invasive breast cancers.

Src family kinases are critical regulators of normal cellular growth, migration, adhesion, and survival (Thomas and Brugge, 1997; Irby and Yeatman, 2000; Frame, 2002; Playford and Schaller, 2004). Aberrant expression or activation of Src family kinases causes perturbation of these activities, leading to transformation and progression of malignant disease (Irby *et al*, 1997, 1999; Dehm and Bonham, 2004; Gallick, 2004). Elevated Src activity has been shown to increase growth rate (Mao *et al*, 1997) and metastatic potential, while inhibiting cell–cell adhesion (Hamaguchi *et al*, 1993) in human cancers. Elevated Src kinase activity has been noted in human breast cancers. Although Src activity may be increased 4- to 20-fold in breast cancer tumours as compared with normal tissues, this increase is not always accompanied by corresponding increases in Src protein expression (Rosen *et al*, 1986; Ottenhoff-Kalff *et al*, 1992; Verbeek *et al*, 1996; Summy and Gallick, 2003). Interaction of Src with numerous breast cancer-associated growth factors and signalling pathways, such as prolactin (Acosta *et al*, 2003), EGFR (Biscardi *et al*, 2000), ERK1/2, PI3-kinase (Gaben *et al*, 2004), and oestrogen receptor (Migliaccio *et al*, 2005), supports the notion that Src activity contributes to the growth and survival of breast cancer cells. Inhibition of Src kinase activity has been shown to inhibit cell growth and migration in both *in vitro* and *in vivo* systems. Using a dominant-negative form of Src, Gonzalez *et al* (2006) demonstrated that interruption of aberrant Src activity in MCF7 cells inhibited the spreading, attachment, proliferation, and migration of these cells *in vitro* while decreasing their tumorigenicity and increasing their rate of apoptosis *in vivo*. A recent study demonstrated that the Src inhibitor SKI-606 blocks the invasion of basal-type MDA-MB-231 breast cancer cells *in vitro*, as well as the spontaneous metastasis of MDA-MB-231 cells from the mammary fat pad of nude mice (Jallal *et al*, 2007).

Dasatinib (BMS-354825, Sprycel) is an orally active Src kinase inhibitor currently approved for imatinib-resistant/intolerant BCR-ABL+ leukaemias. Dasatinib is currently under evaluation in phase II clinical trials for solid tumours. Known chemically as [N-(2-chloro-6-methylphenyl)-2-(6-(4-(2-hydroxyethyl)piperazin-1-yl)-2-methylpyrimidin-4-ylamino)thiazole-5carboxamide], dasatinib inhibits Abl and Src family kinases at low concentrations (IC_50_ <1.0 nM). At higher concentrations, dasatinib may inhibit other tyrosine kinases such as p38, Akt, FAK, and the receptor tyrosine kinases PDGFR, c-kit, and Ephrin (Lombardo *et al*, 2004). This drug also has shown antiproliferative effects in lung and prostate tumour cell lines at low nanomolar concentrations (Lombardo *et al*, 2004; Nam *et al*, 2005; Song *et al*, 2006; Park *et al*, 2008). Recently, Finn *et al* (2007) demonstrated the selective efficacy of dasatinib in ‘triple-negative’ breast cancer cells, which lack oestrogen receptor, progesterone receptor, and HER2 (Boggon and Eck, 2004). However, the molecular pathways and downstream effects of dasatinib in breast cancer cells have not been investigated earlier.

The cytostatic and antimetastatic properties of dasatinib make it a promising component of multi-drug regimens when paired with cytotoxic agents such as anthracyclines. In this study, we evaluated the effects of dasatinib alone, and in combination with doxorubicin, on the proliferation, cell cycle distribution, viability, and invasive capacity of select breast cancer cell lines. We found that dasatinib inhibits both cell cycle progression and invasiveness in sensitive cells. Furthermore, the combination of doxorubicin and dasatinib synergistically decreased cell metabolism, proliferation, and viability in the dasatinib insensitive MCF7 cell line, lowering the IC_50_ of doxorubicin by more than one log unit. Additionally, combination treatment of dasatinib-sensitive MDA-MB-231 cells produced a stronger inhibition of migration and invasion than with either drug alone. These results indicate that dasatinib synergizes with the anthracycline doxorubicin to kill a variety of breast cancer cell lines, and provides a rationale for including dasatinib as a cytostatic, anti-invasive agent in multi-drug regimens for primary breast cancers.

## Materials and methods

### Cell culture

MDA-MB-231, MCF7, and T47D cell lines were obtained from the ATCC by Drs Gordon Mills and Janet Price (MD Anderson Cancer Center, Houston, Texas, USA). All cell lines were grown at 37°C and in 5% CO_2_. MDA-MB-231 and T47D cells were maintained in DMEM/F12 media (Invitrogen, Carlsbad, CA, USA) supplemented with 10% fetal calf serum (Hyclone, Logan, UT, USA), 100 U/ml penicillin, and 100 *μ*g/ml streptomycin. MCF7 cells were maintained in MEM media (Invitrogen) supplemented with 10% fetal calf serum, 100 U/ml penicillin, 100 *μ*g/ml streptomycin, 2 mM L-glutamine, 0.1 mM non-essential amino acids, 1 mM sodium pyruvate, and MEM vitamin solution.

### Antibodies and reagents

Dasatinib was provided by Bristol-Myers Squibb Pharmaceutical Research Institute (Princeton, NJ, USA) and kept dissolved in DMSO at −20°C. Doxorubicin was purchased from Sigma (St Louis, MO, USA). Commercially available antibodies used were: p38 MAPK, CrkL, Akt, phospho-Src (Y416), phospho-Src (Y527), phospho-p130^CAS^ (Y410), phospho-FAK (Y576), phospho-p38 MAPK (T180/Y182), phospho-Abl (Y245), phospho-CrkL (Y207), phospho-Akt (S473), phospho-Erk1/2 (T202/Y204), and phospho-Akt-substrate (RXRXXS/T) from Cell Signaling (Danvers, MA, USA); c-Src, p130^CAS^, Abl, Erk1/2, p27^Kip1^, and actin from Santa Cruz Biotechnology (Santa Cruz, CA, USA); cortactin and phospho-tyrosine (4G10) from Upstate Biotechnology (Billerica, MA, USA); p21^WAF1^ from BD Biosciences (San Jose, CA, USA) and AlexaFluor488-phalloidin and Cy3-anti-mouse from Invitrogen; phospho-N-WASP (Y256) and N-WASP from ECM Biosciences (Versailles, KY, USA).

### Viability and proliferation assays

For cell counting and trypan blue exclusion, cells were grown in the appropriate media plus dasatinib for up to 72 h, collected by scraping, diluted in trypan blue dye, and counted with a Brightline hemocytometer (Hausser Scientific, Horsham, PA, USA). Proliferation was determined using an MTT assay (#M5655, Sigma-Aldrich). Cells were seeded at a density of 3–5 × 10^3^ cells per well of 96-well plate with complete medium 24 h prior to treatment. Cells were then treated for 24, 48, and 72 h before MTT reagent was added and absorbance was read at 570 nm, per manufacturer's instructions.

### Cell cycle and cell death assays

Cell cycle distribution was analysed by plating cells at a density of 3–4 × 10^5^ cells per 10 cm culture dish with complete medium 24 h prior to treatment. Cells were then treated with dasatinib for 48 h, collected, fixed in 70% ethanol, washed, and stained with a 5% propidium iodide solution. Samples were detected with a FACSCalibur (Becton Dickinson, Franklin Lakes, NJ, USA) and analysed with FlowJo software (Tree Star Inc., Ashland, OR, USA). Annexin-V and propidium iodine staining were performed with a flow cytometric apoptosis detection kit (BD Pharmingen, San Jose, CA, USA) per the published manual and analysed by FACSCalibur. BrdU and 7-AAD staining and analyses were performed per the manufacturer's published protocols (FITC Flow Kit, BD Biosciences).

### Immunoprecipitation and Immunoblotting

Cells were grown in complete media overnight, treated with dasatinib for 2–48 h, washed, collected by scraping, and lysed in 1% NP-40 buffer supplemented with the appropriate proteinase and phosphatase inhibitors. Protein concentrations were determined by a Bradford assay and equal amounts of each sample were either prepared for loading or immunoprecipitated overnight with the appropriate antibody. Samples were then incubated with Protein A-Sepharose beads (Sigma), pelleted by centrifugation, washed and released by boiling in Laemmli sample buffer. Western blot analysis was performed with whole cell lysates or immunoprecipitated samples resolved by SDS–PAGE and transferred onto Immobilon-P Transfer Membranes (Millipore Corp, Billerica, MA, USA). The membranes were blocked overnight with blocking buffer (5% milk or 5% BSA, depending on the antibody, with 0.1% Tween-20). The blots were incubated with primary and then secondary antibodies for 1 h each at room temperature. Immunoreactive bands were visualised by enhanced chemiluminescence (Amersham, Piscataway, NJ, USA). Membranes were then stripped for 30 min at 37°C using Stripping buffer (Pierce, Rockford, IL, USA), reblocked, and probed for actin, GAPDH, or the non-phosphorylated protein being analysed as loading controls. Densitometric analysis was performed using the NIH software, ImageJ (Macintosh platform, Bethesda, MD, USA), to determine the ratio of phosphorylated protein to total protein. For Src-pY416:c-Src, IC_50_ values were calculated based on exponential regressions of the plotted ratios using Microsoft Excel. For IC_50_ values of Src inhibition, ratio values of Src-pY416 to total Src were generated by exponential regression (Microsoft Excel).

### Immunofluorescence

Cells were grown and treated on glass chamber slides or glass coverslips, fixed in 4% paraformaldehyde, permeabilised with 0.1% Triton X-100, blocked in 1% BSA, and stained with anti-α-tubulin (Sigma) and Alexa-488 fluorescent phalloidin (Molecular Probes, Carlsbad, CA, USA). Tubulin staining was detected with a Cy3-conjugated donkey anti-mouse antibody (Jackson ImmunoResearch, West Grove, PA, USA). Slides were prepared using ProLong Antifade mounting media (Molecular Probes), and imaged with a Nikon Eclipse TE2000U microscope and MetaMorph imaging software (Molecular Devices, Toronto, Canada). Invadopodia were analysed by seeding cells on a thin layer of FITC-labeled gelatin (VWR, West Chester, PA, USA) as described earlier (Clark *et al*, 2007). Cells were allowed to invade for 20 h, and the slides were then processed as described above, stained first for cortactin and then with Cy3-anti-mouse. Invadopodia were counted from 10 random fields in each sample (blinded) and averaged.

### Cell migration and invasion assays

Migration was measured by wound healing assay, in which cells were grown to 80% confluence in 6-well plates, streaked with a sterile pipette tip, and allowed to recover in dasatinib-treated media. After 6 h, plates were visualised at × 10 magnification and migration determined by measuring wound width (in pixels) using the MetaMorph imaging software (Molecular Devices, Toronto, Canada). Invasiveness was determined using Matrigel invasion chambers with an 8 *μ*m-pore membrane (BD Biosciences) seeded with 2.5 × 10^4^ cells each. Dasatinib-treated media was used in both the upper and lower chambers, with serum added only to the lower chamber. Cells were allowed to invade for 24 h through the Matrigel, at which point the inserts were removed and the membranes scrubbed and fixed in methanol. Invading cells were then stained and mounted on slides with Prolong Antifade with DAPI (Molecular Probes). Membranes were then visualised with an epifluorescent microscope (Nikon, Tokyo, Japan), and quantification of invading cells (visualised as DAPI-stained nuclei) was determined in six random fields per sample.

### Statistics

Descriptive statistics including mean values and s.d. were calculated using Microsoft Excel or Prism software (GraphPad, La Jolla, CA, USA). Statistical significance was determined by two-sample student *t*-tests (*P*=0.05). Calculation of GI_50_ (Dm) values, measures of sigmoidicity (m), correlation coefficients (*r*), and combination indices (CI) of multi-drug treatments were performed using the CalcuSyn software (Biosoft, Cambridge, UK). Degree of cooperation between dasatinib and doxorubicin was determined from the combination index (CI) as follows: CI>1 indicates antagonism; CI=1 indicates additivity; 1>CI>0.3 indicates synergy; 0.3>CI>0.1 indicates strong synergy (Chou, 1976; Chou and Talalay, 1981).

## Results

### Dasatinib inhibits proliferation and metabolism of sensitive MDA-MB-231 cells

**Figure 1:**
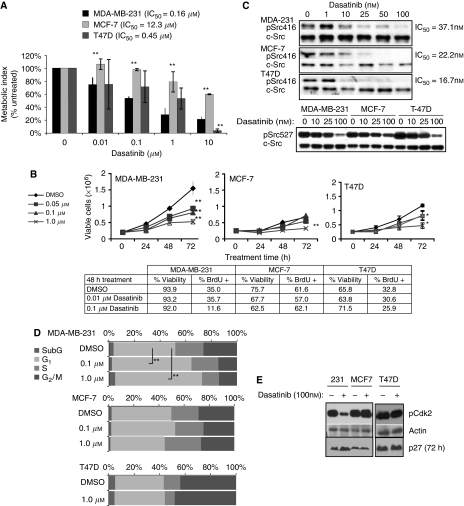
Inhibition of cell growth and G1 progression in dasatinib-sensitive breast cancer cells. (**A**) Cells were grown in 96-well plates with complete medium, then treated with dasatinib for 48 h before analysis by MTT assay. Results are shown as a percent of DMSO-treated samples and represent the mean of four independent assays with 4–5 replicates each. Error bars are standard error of the mean (s.e.m.). Asterisks denote significance in comparison to MDA-231 sample within each treatment dose. (**B**) Cells were plated in 6-well dishes with complete medium and treated with dasatinib for 48 h. Samples were collected at indicated time points and counted with a hemocytometer. Viability was determined by trypan blue exclusion. BrdU positivity was analysed by flow cytometry. (**C**) Cells were treated with DMSO or dasatinib for 2 h and whole cell lysates probed for phospho-Src (Y416 or Y527), stripped, and reprobed for c-Src. Response for each dose was calculated as the ratio of phospho-Src to total-Src. IC_50_ was calculated by best-fit regression of data points. (**D**) Cells were treated for 48 h with DMSO, 0.1 or 1.0 *μ*M dasatinib, fixed, and stained with propidium iodide, and analysed by flow cytometry for cell cycle distribution. (**E**) Whole lysates of cells treated with 100 nM dasatinib for 2 h (72 h for p27 blot, as indicated) were probed for phospho-Akt substrate, actin, and p27. Results are representative of at least three independent experiments. ^*^Indicates *P*<0.05; ^**^indicates *P*<0.01.

**Figure 2:**
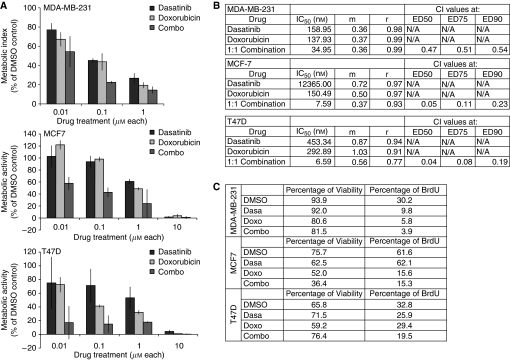
Synergistic growth inhibition by dasatinib–doxorubicin combinations. (**A**) Cells were grown in 96-well plates with complete medium and treated with dasatinib alone, doxorubicin alone, or both drugs in combination (1 : 1 ratio) for 48 h before analysis by MTT assay. Metabolic rates are shown as percentages of the control (DMSO)-treated sample. (**B**) IC_50_ values for each drug (Dm) and combination indexes (CIs) were calculated by logarithmic regression analysis with CalcuSyn software (BioSoft, Cambridge, UK). CI values less than 1 indicate synergy between dasatinib and doxorubicin. Results are shown as the mean of five replicates and are representative of at least two independent experiments. (**C**) Cells were treated 48 h with either drug alone or in combination (1 : 1 ratio). BrdU was added for last 1 h of culture before cells were collected and assessed for viability (by trypan blue exclusion) and cell cycle distribution. 7-AAD was used to stain total DNA. BrdU positivity represents cells actively replicating in the last hour of culture. ^**^Indicates *P*<0.01.

Because the molecular mechanism of action of dasatinib against breast cancer cells has not been studied earlier, we examined the effects of dasatinib on three commonly studied breast cancer cell lines, MDA-MB-231, MCF7, and T47D, which were previously characterised as highly sensitive (MDA-MB-231) and moderately resistant (MCF7, T47D) to dasatinib (Finn *et al*, 2007). To compare dasatinib-induced inhibition of metabolic activity, cells were treated with increasing doses of dasatinib for 48 h and metabolic activity was quantified by MTT assay. The IC_50_ for metabolic inhibition was calculated from these data (Figure 1A). MDA-MB-231 cells demonstrated the strongest inhibition with a calculated IC_50_ of 0.16 *μ*M. MCF7 and T47D cells were less responsive, with IC_50_ doses of 12.3 and 0.45 *μ*M, respectively. Cell counts taken over 72 h of treatment with 100 nM dasatinib demonstrated an increase in the doubling time of MDA-MB-231 cells, whereas MCF-7 cell proliferation was less affected (Figure 1B). Dasatinib treatment of MDA-MB-231 cells achieved significant inhibition (*P*<0.01) of cell proliferation at 0.05 *μ*M, whereas MCF7 did not exhibit significant inhibition with doses lower than 1.0 *μ*M. IC_50_ doses for dasatinib calculated from cell counts after 72 h of dasatinib treatment demonstrated a three-fold difference between MDA-MB-231 and MCF7 (0.33 *μ*M and 0.99 *μ*M, respectively). BrdU uptake by replicating MDA-MB-231 cells was also significantly reduced from 35% (DMSO control) to 12%. IC_50_ values calculated from the uptake of BrdU demonstrated the same trend, in which MDA-MB-231 are the most sensitive and MCF7 the most resistant. To test for cell death due to dasatinib treatment, cells were treated with dasatinib and percent viability after 48 h of treatment was determined by trypan blue exclusion. No decrease in cell viability was observed in MDA-MB-231 cells, although a slight decrease in viability was noted in MCF7 cells (Figure 1B). The absence of dasatinib-induced apoptosis in MDA-MB-231 cells was confirmed by flow cytometric analysis of Annexin-V and PI staining (data not shown). Furthermore, no increase in PARP cleavage was detected in lysates of MDA-MB-231 or T47D cells after 48 h of treatment (Figure 2C).

### The degree of Src inhibition does not correlate with proliferative inhibition by dasatinib

Given the wide variation in dasatinib sensitivity across breast cancer cell lines, we examined whether Src inhibition was a useful biomarker for dasatinib response. The IC_50_ values for Src inhibition were calculated from the dose-dependent dephosphorylation of tyrosine 416, which is an activating phosphorylation site within Src (Figure 1C). We observed that, despite their wide variability in metabolic response, all three cell lines exhibited comparable inhibition Src activity. Within 2 h, Src phosphorylation was attenuated by >75% in all cell lines following treatment with 100 nM dasatinib, which is an achievable dose in clinical trials (Copland *et al*, 2006). Interestingly, the IC_50_ values for Src inhibition, which ranged between 16.7–37.0 nM, were much lower than the doses required for 50% inhibition of metabolic activity. As an additional measure of Src activation, we also examined the phosphorylation state of Src on tyrosine 527 (Y527), which is an inhibitory phosphorylation site. Dephosphorylation of Y527 was also observed with dasatinib treatment (Figure 1C), although at much higher concentrations (10-fold) than required for 50% inhibition of the activating (Y416) site phosphorylation. This may indicate a non-specific inhibition of the activity of the C-terminal Src kinase, Csk, at high doses of dasatinib (Roskoski, 2005; Lieser *et al*, 2006).

We also examined basal levels of phospho-Src (Y416) across a panel of breast cancer cell lines to determine whether basal Src activity was a predictor of dasatinib sensitivity. Basal phospho-Src (Y416) levels were not dramatically different between these three lines (Supplementary Figure 1). Interestingly, we found that the weakly invasive MDA-MB-468 cell line had very high levels of basal Y416 phosphorylation. However, despite the strong basal phosphorylation in these cells, phospho-Y416 was still completely inhibited by 100 nM dasatinib treatment (Supplementary Figure 1) and the cells were calculated to have an IC_50_ for metabolic activity of more than 10 *μ*M. This indicates that neither basal Src activity nor loss of Y416 phosphorylation following dasatinib treatment are adequate predictors of dasatinib sensitivity. We also considered whether other kinases known to be inhibited by dasatinib were relevant markers of dasatinib effectiveness in these breast cancer cell lines. In this regard, dasatinib has been reported to block the activities of c-kit, PDGFR and the ephrin receptor (EphA2) at similar doses required for Src inhibition *in vitro* (Lombardo *et al*, 2004; Chen *et al*, 2006; Schittenhelm *et al*, 2006; Wang *et al*, 2007). However, c-kit and PDGFR were not expressed in the dasatinib-sensitive MDA-MB-231 cell line (Supplementary Figure 1) (Hines *et al*, 1995; Meric *et al*, 2002). On the other hand, although we observed that the ephrin receptor was highly expressed in the sensitive MDA-MB-231 cells, dasatinib did not alter its tyrosine phosphorylation (data not shown) (Zelinski *et al*, 2001). These data indicate that c-kit, PDGFR, and Ephrin receptor, known targets of dasatinib, were not effective markers for dasatinib sensitivity in these breast cancer cell lines. This does not exclude the possibility that unknown targets of dasatinib are being inhibited when using high doses of the drug.

### Dasatinib treatment induces G_1_ arrest in MDA-MB-231 cells

Owing to the inability of dasatinib to induce cell death, we reasoned that the observed inhibition of proliferation could result from a block in cell cycle progression. Dasatinib-induced cell cycle arrest has not been investigated in solid tumours. Therefore, cell cycle distribution was measured after 48-h dasatinib treatment (Figure 1D). MDA-MB-231 cells demonstrated a significant increase in the proportion of cells in G_1_ phase after 1 *μ*M dasatinib treatment (64 *vs* 41%; *P*<0.001). MCF7 and T47D cells, however, showed no significant change in cell cycle distribution (Figure 1D). No significant accumulation of sub-G_1_ cells was observed, confirming that dasatinib did not induce cell death. To verify G_1_ arrest in MDA-MB-231 cells, we investigated the status of cell cycle regulatory proteins Cdk2 and p27^Kip1^ after dasatinib treatment (Sherr and Roberts, 1999). Cdk2 phosphorylation on an activating site was sharply decreased after 100 nM of dasatinib in MDA-MB-231 cells, but not in MCF7 or T47D cells. In addition, p27 accumulation, an indicator of cell cycle arrest, was observed only in the MDA-MB-231 cells after 72 h of dasatinib treatment (Figure 1E).

### Combination treatment of dasatinib and doxorubicin synergistically inhibits metabolism and growth

**Figure 3:**
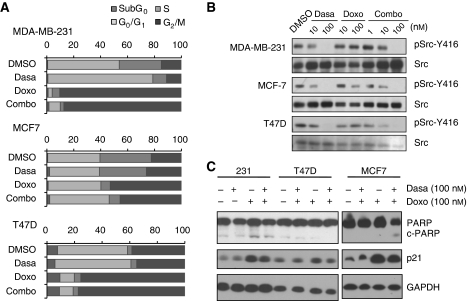
Effects of combination treatment on cell cycle progression and phospho-Src. (**A**) Cells were treated 48 h with either drug alone or in combination (1 : 1 ratio), then collected and assessed for cell cycle distribution by 7-AAD staining. Flow cytometry was used to analyse staining of fixed cells. (**B**) Protein lysates of MDA-MB-231, MCF7, and T47D cells treated for 2 h were immunoblotted as indicated for pSrc-Y416, stripped and reprobed for total Src. (**C**) Cells were treated for 48 h with DMSO, 100 nM dasatinib, 100 nM doxorubicin, or 100 nM of each drug. Whole lysates were immunoblotted for PARP, p21^WAF1^, and GAPDH.

Dasatinib is likely to be used in combination with traditional chemotherapeutics, so we examined whether combination treatment with a cytotoxic agent such as doxorubicin could heighten the sensitivity to either drug. Dose–response curves were generated to investigate the synergistic effects of combination therapy with dasatinib and doxorubicin on cell proliferation using an MTT assay (Figure 3A). In MDA-MB-231 cells, dasatinib treatment alone inhibited proliferation at an IC_50_ of 160 nM, whereas doxorubicin alone blocked proliferation at an IC_50_ of 140 nM (Figure 3B). On the other hand, treatment with a 1 : 1 drug combination (simultaneous administration) inhibited proliferation at a much lower IC_50_ of 35 nM for each drug. Calculation of the combination index (CI), in which values below 1 indicate synergy, demonstrates moderate synergism of the two drugs (CI=0.47). Interestingly, the synergistic effects of the two drugs were considerably stronger in MCF7 and T47D cells, where dasatinib alone had very weak effects on proliferation. The CI values calculated for 50% inhibition of these cells were 0.05 and 0.04, respectively, indicating very strong synergy. In the most dasatinib-resistant cell line, MCF7 (IC_50_=12 *μ*M), combination treatment of the drugs in a 1:1 ratio inhibited cell growth by 50% at 7.6 nM of each drug. Similarly in T47D cells, 50% inhibition of cell growth was achieved at 6.6 nM of each drug. These values represent a dramatic decrease in the dosage of either dasatinib or doxorubicin required for comparable levels of growth inhibition with either drug alone. Specifically, in MCF7 cells the dose of doxorubicin needed for 50% growth inhibition can be decreased by 95% (from 150 to 7.6 nM) when given in combination with an equal dose of dasatinib. Similarly, in T47D cells the IC_50_ for doxorubicin can be decreased by 98% (293–6.6 nM). These changes represent significant improvements in the efficacy of a currently used chemotherapeutic agent, doxorubicin, in blocking proliferation in these cells.

We then examined whether the synergistic effect of dasatinib and doxorubicin on growth inhibition was the result of altered rates of cell cycle progression or apoptosis. In MDA-MB-231 cells, viability was not greatly affected by any drug combination. However, addition of dasatinib to the doxorubicin treatment did reduce the population of BrdU-positive cells from 5.8 to 3.9% (Figure 3C). Analysis of cell cycle distribution by flow cytometry confirmed that there was a significant decrease in the percentage of cells in S phase (Figure 2A). We also observed that the doxorubicin-mediated G_2_/M arrest was predominant over dasatinib-induced G_1_ arrest in cells treated with the drug combination, such that 82.6% of live cells were in G_2_/M phase after 48 h of treatment (Figure 2A). This was not significantly different than G_2_/M accumulation in cells treated with doxorubicin alone (82.1% in G_2_/M). Consistent with the dominant G_2_/M arrest in the doxorubicin-treated cells, similar levels of accumulation of p21^WAF1^ were observed after treatment with either doxorubicin alone or dasatinib plus doxorubicin (Figure 2C). These data indicate that co-treatment with dasatinib did not significantly alter the effects of doxorubicin on cell cycle progression or cell viability in MDA-MD-231 cells.

On the other hand, in MCF7 cells the combination treatment did enhance cell killing, reducing viability from 52.0% with doxorubicin treatment alone to 36.4% with the combination treatment. This may contribute to the strong synergy between dasatinib and doxorubicin seen in the MCF7 cells. Importantly, as with dasatinib treatment alone, there was no increase in PARP cleavage after combination treatment (Figure 2C), suggesting that the increase in cell death was the result of a non-apoptotic mechanism. To determine whether the synergy between dasatinib and doxorubicin resulted from differential effects on Src activation, the level of Src (Y416) phosphorylation in MDA-MB-231 and MCF7 cells was examined. However, combination treatment did not induce additive inhibition of Src in either cell type (Figure 2B). Taken together, these results suggest that the synergy between doxorubicin and dasatinib in MDA-MB-231 and MCF7 cells results from inhibition of distinct pathways, neither of which can be accounted for by altered sensitivity to Src inhibition.

### Dasatinib-sensitive cells undergo cytoskeletal contraction

**Figure 4:**
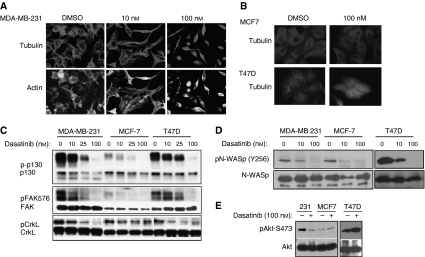
Dasatinib treatment disrupts cytoskeletal organisation and cellular invasion. (**A**, **B**) MDA-MB-231 (**A**), MCF7, and T47D (**B**) cells were grown on glass chamber slides and treated with dasatinib for 48 h before staining with antitubulin antibody and FITC-phalloidin. (**C**) MDA-MB-231, MCF7, and T47D cells were treated with increasing doses of dasatinib (DMSO, 10, 25, or 100 nM) for 2 h. Whole cell lysates were immunoblotted for phospho -FAK(Y576), -p130 (Y410) and -CrkL (Y207) . Membranes were then stripped and reprobed for total p130, FAK, or CrkL, respectively. (**D**) Cells were probed for phospho-N-WASP (then reprobed for total N-WASP) after treatment with DMSO, 10, or 100 nM dasatinib for 2 h. (**E**) DMSO- or dasatinib-treated cells were lysed and probed for phospho-Akt (S473). The membrane was then stripped and reprobed for total Akt as a control. All images are representative of at least three independent experiments.

The mesenchymal-like MDA-MB-231 cells normally grow as flattened, spindle-shaped cells, characterised by many filopodia-like processes. Upon dasatinib treatment, however, the cells displayed a very different morphology that was characterised by a round, contracted appearance. As seen in Figure 4A, the actin and tubulin cytoskeletal structure of the cells was disrupted, creating a dense and compact cell body in which the intricate actin branching structures appeared to have collapsed and filamentous tubulin is compacted. On the other hand, only minor changes in morphology were observed in the MCF7 and T47D (Figure 4B) cells treated with a dasatinib concentration (100 nM) that clearly inhibited Src activation (Figure 1C). This is consistent with the reduced effectiveness of dasatinib on the growth of these cells.

To determine whether inhibition of cytoskeletal regulatory proteins might predict dasatinib-sensitivity, cells were treated with dasatinib and protein lysates probed for phosphorylation of the cytoskeletal regulatory proteins focal adhesion kinase (FAK), p130^CAS^, and CrkL, each of which are substrates of Src family kinases (Gonzalez *et al*, 2006). In MDA-MB-231 and T47D cells, we observed that dasatinib treatment strongly inhibited FAK phosphorylation at the activating site Y576. MCF7 cells exhibited low basal phospho-FAK (Y576), which was also comparably affected (Figure 4C). Active FAK phosphorylates N-WASP, a Cdc42 effector protein that promotes branched actin polymerisation, at Y256. Dasatinib treatment also inhibited N-WASP phosphorylation at Y256 in MDA-MB-231, MCF7, and T47D cells (Figure 4D). Although more basal N-WASP phosphorylation was present in the T47D cells (compared with MDA-MB-231 cells, data not shown), residual phosphorylation after dasatinib treatment was comparable. In addition, dasatinib treatment induced a dose-dependent reduction in the phosphorylation of p130^CAS^, which was consistent for all three cell lines (Figure 4C). While basal activity of CrkL was comparable in all three lines, dephosphorylation of CrkL (Y207) was only evident in MDA-MB-231 and T47D cells, with no significant change in MCF7 lysates. Given the high basal CrkL phosphorylation in all three cell lines and the selective dephosphorylation of CrkL only in dasatinib-sensitive cells, decreased CrkL tyrosine phosphorylation appeared to most accurately reflect dasatinib sensitivity. Thus, CrkL dephosphorylation on Y207 may serve as an effective biomarker for dasatinib response in future studies.

Because Src can positively regulate Akt through PI3K, we investigated whether phospho-Akt may also be a marker for dasatinib sensitivity. Consistent with the characteristics of mesenchymal-like tumours, MDA-MB-231 cells exhibited a high level of basal Akt activity. Dasatinib treatment strongly inhibited the phosphorylation of Akt at serine 473 in MDA-MB-231 cells, indicative of decreased Akt activity. MCF7 cells, which retain a more epithelial phenotype, had lower levels of basal Akt (S473) phosphorylation that were not affected by dasatinib treatment. T47D cells also did not demonstrate any loss in pAkt with dasatinib treatment. Because we did not observe any increase in apoptosis with dasatinib treatment, it is unlikely that dasatinib-inhibition of Akt is affecting cell survival pathways. Previous studies have suggested a role for Akt in the regulation of cell cycle progression via phosphorylation of Cdk2 and p27^Kip1^ (Shin *et al*, 2002; Grille *et al*, 2003; Maddika *et al*, 2008), which were inhibited by dasatinib only in the MDA-MB-231 cells (Figure 1E). Thus, the dephosphorylation of Akt and its downstream substrates in MDA-MB-231 cells following dasatinib treatment suggests that phosphorylation of these proteins may serve as indirect markers for dasatinib efficacy.

### Migration and invasion are inhibited by dasatinib treatment

**Figure 5:**
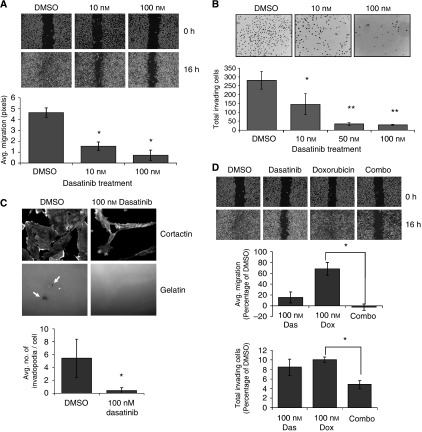
Doxorubicin and dasatinib synergize to block migration and invasion of MDA-MB-231 cells. (**A**) MDA-MB-231 cells were grown to 80% confluence in glass chamber slides and treated for 6 h before being streaked with a sterile pipette tip. Phase contrast images were taken at 0 and 6 h after wounding. All bright field images were obtained at × 10 magnification and wound width quantified (in pixels) using the NIH Image J software. (**B**) Cells were pre-treated with dasatinib for 4 h before transfer to Matrigel invasion chambers in serum-free, dasatinib-containing media. Cells were allowed to invade for 16 h towards media containing 10% FCS plus dasatinib. Data represent the average number of invading cells per membrane. (**C**) MDA-MB-231 cells were plated on FITC-gelatin in media containing DMSO or 100 nM dasatinib and allowed to adhere and invade for 20 h. Cells were then stained for cortactin, and invadopodia counted as co-localisations of cortactin staining and degraded FITC-signal (indicated by white arrows) in 10 random fields per sample. Results represent the average number of invadopodia per cell. (**D**) Wound healing and Matrigel invasion assays were repeated as in panels A and B, with dasatinib-, doxorubicin-, or combination (100 nM each drug)-treated cells. Error bars represent s.d. ^*^Indicates *P*<0.05; ^**^indicates *P*<0.01.

Based on the observed morphology changes in MDA-MB-231 cells following dasatinib treatment, we investigated the functional effects of dasatinib on cell migration and invasion. MCF7 and T47D cells were not tested because they are resistant to migration along the edge of a wound, are not invasive, and responded poorly to dasatinib. To measure effects on cell migration we used streak assays, where the ability of cells to repopulate a wounded area in a monolayer can be measured. We observed that untreated MDA-MB-231 cells were highly motile and repopulated the denuded area within 24 h. However, following dasatinib treatment, migration into the wounded area was greatly abrogated with a clear dose-dependent association (Figure 5A, *P*<0.05). To measure cell invasion, we used a Boyden chamber assay with Matrigel-coated filters. In these assays invasion through the Matrigel by MDA-MB-231 cells was decreased by 88% with 50 nM dasatinib treatment (Figure 5B; *P*<0.01). To confirm that these effects were due to cytoskeletal defects and not a result of inhibited cell proliferation, we studied the formation of invadopodia in individual invading cells. Invadopodia are actin-rich, finger-like projections that protrude into the extracellular matrix, secreting proteinases to degrade the matrix and allow for invasion of a cell through the barrier (Weaver, 2006). By plating MDA-MB-231 cells on a fluorescently-labeled gelatin substrate, we are able to visualise discrete points of matrix degradation and cytoskeletal extensions, which are identified as active invadopodia (Clark *et al*, 2007). Dasatinib treatment reduced the average number of invadopodia per cell from 5.4 to 0.4, indicating a near complete blockage of cytoskeletal remodelling activity and invasive capacity (Figure 5C; *P*<0.05).

We then determined whether doxorubicin and dasatinib synergized to block the migratory and invasive capacity of MDA-MB-231 cells. Cells were treated with 100 nM dasatinib, alone or in combination with an equimolar dose of doxorubicin. In these assays, doxorubicin treatment alone only weakly affected the migration of MDA-MB-231 cells. However, in combination with dasatinib, the antimigratory effect was significantly stronger (Figure 5D; *P*<0.05). Because the wound assay is performed at a short time point (6 h), effects on proliferation cannot account for this difference. In the Matrigel invasion assay, doxorubicin treatment alone reduced invasion of MDA-MB-231 cells to a level near that achieved with an equal dose of dasatinib. Importantly, in combination treatment, the level of invasion was further reduced (Figure 5D; *P*<0.05). These data indicate that combination treatment with dasatinib and doxorubicin elicited greater effects on migration and invasion than either drug alone, and further support a strategy where breast cancers may be more effectively treated by combining traditional chemotherapeutics with dasatinib.

## Discussion

Src family kinases play critical roles in the progression and survival of human cancers. Thus we tested whether dasatinib, an effective Bcr-Abl and Src inhibitor, blocked breast cancer cell proliferation. For these studies, we evaluated the sensitivity of three commonly used breast cancer cell lines, MDA-MB-231, MCF7, and T47D, to dasatinib treatment and investigated the resulting molecular effects. Because cellular response to dasatinib did not correlate with inhibition of Src kinase activity, we studied the differential signaling pathways that may mediate dasatinib sensitivity. Phosphorylation of Src on tyrosine 416, an activating autophosphorylation site, was consistently inhibited in all cell lines. Although some loss of phosphorylation at the inhibitory phosphorylation site tyrosine 527 (Boggon and Eck, 2004) was observed with high doses of dasatinib (100 nM), this was also consistent across the cell lines tested and did not correlate with changes in proliferation. Other potential dasatinib targets, such as PDGFR and c-kit, were not present in these cell lines. Ephrin receptor was present but not inhibited by dasatinib. Dasatinib also targets Abl at low concentrations, and the presence of active Abl in MDA-MB-231 has been previously suggested. However, we were unable to detect basal phosphorylation of Abl in any of the breast cancer cell lines we tested (data not shown).

Given the relatively uniform inhibition of Src, we sought to identify other potential markers that may predict sensitivity to dasatinib. Previous studies have examined gene expression arrays to identify potential markers for dasatinib sensitivity and found that caveolin and moesin mRNAs are downregulated in dasatinib-sensitive cells after treatment (Finn *et al*, 2007; Huang *et al*, 2007). However, we did not detect any change in the expression of either protein after 48 h of dasatinib treatment in the dasatinib-sensitive MDA-MB-231 cells (data not shown), indicating that at the protein level these gene products are unlikely to serve as useful markers. On the other hand, we observed that phosphorylation of the Src family kinase substrate CrkL may serve as an effective biomarker of dasatinib responsiveness. CrkL is an adaptor protein associated with adhesive properties of cells, and is phosphorylated on Y207 by Src family kinases. Dasatanib reduced phospho-CrkL (Y207) levels by 76% in the sensitive MDA-MB-231 cells, while phospho-CrkL (Y207) decreased by only 17 and 34% in the relatively insensitive MCF7 and T47D cells, respectively. Based on these data, phosphorylation of CrkL on Y207 may serve as a biomarker for dasatinib response in breast cancer cells.

Dasatinib also strongly inhibited Akt phosphorylation at S473 in the MDA-MB-231 cells, presumably through downregulation of the PI3K pathway resulting from decreased Src activity (Lu *et al*, 2003). Although dasatinib can directly inhibit Akt kinase activity (Lombardo *et al*, 2004), we observed significant loss of Akt phosphorylation at 100 nM, a dose far below the reported IC_50_ for Akt of greater than 50 *μ*M. Because only the dasatinib-sensitive MDA-MB-231 cells showed a reduction in Akt phosphorylation following dasatinib treatment, decreases in the phosphorylation of Akt or its downstream substrates may also serve as predictive markers for dasatinib response.

An important observation of these studies is that dasatinib treatment induced severe morphological and functional changes in the sensitive MDA-MB-231 cells. These observations were consistent with the observed reduction in the phosphorylation of cytoskeletal regulators such as CrkL. Disruption of cytoskeletal architecture upon treatment resulted in more compact, rounded cells that lacked the extensively branched actin structures normally seen in MDA-MB-231 cells. Consequently, migration and invasion were also dramatically impaired. Dasatinib-treated cells were unable to re-infiltrate the denuded area in scratch assays. *In vitro* invasion of MDA-MB-231 cells was also inhibited by >90%, and invadopodia formation was almost completely blocked. These results indicate that the antimetastatic properties of dasatinib may be mediated by impaired actin polymerisation and cytoskeletal control. Many factors affect invasiveness, and it cannot be excluded that the secretion of matrix-degrading enzymes such as MMP-9 may also be impaired in dasatinib-treated cells, contributing to the loss of invasion. It is likely however, that the severity of the cytoskeletal defect in MDA-MB-231 and loss of invadopodia formation, are sufficient to cause the decrease in invasion seen with dasatinib treatment.

Vigneron *et al* (2005) demonstrated earlier that aberrant Src signaling impaired the p21^WAF1^-mediated senescence induced by the anthracycline doxorubicin. Because of these findings, we investigated whether Src inhibition can strengthen the effects of doxorubicin. Importantly, we observed that dasatinib sensitises cells to the growth arrest and cell killing induced by doxorubicin. *In vitro* experiments with MCF7 and T47D cells, which are refractory to inhibition by dasatinib alone, demonstrated that doxorubicin doses could be reduced 20- to 44-fold in each cell line when combined with an equivalent dose of dasatinib to maintain the same metabolic inhibition observed with doxorubicin alone. These levels are below the maximal plasma concentrations of dasatinib achieved in Phase I clinical trials, and are therefore clinically relevant (Copland *et al*, 2006). Furthermore, combination treatment of MDA-MB-231 cells demonstrated an additive inhibition of migration and invasion, suggesting that adding dasatinib to a doxorubicin therapy may further reduce the risk of metastasis. It is important to note that our studies were performed using simultaneous administration of dasatinib and doxorubicin in equimolar doses. Modification of the dosing schedule or drug ratio may further improve the cooperative actions between these two compounds. Nevertheless, these results provide strong support for the idea of using dasatinib as an effective addition to multi-drug regimens with traditional chemotherapeutics and warrant future investigation.
