# Supplementary material for: Dasatinib synergizes with doxorubicin to block growth, migration, and invasion of breast cancer cells
Source: Br J Cancer. 2009 Jun 9;101(1):38–47. doi: 10.1038/sj.bjc.6605101 (PMC2713704; doi:10.1038/sj.bjc.6605101)
Supplement: Supplementary Figure 1 [file 6605101x1.ppt]

## Slide 1
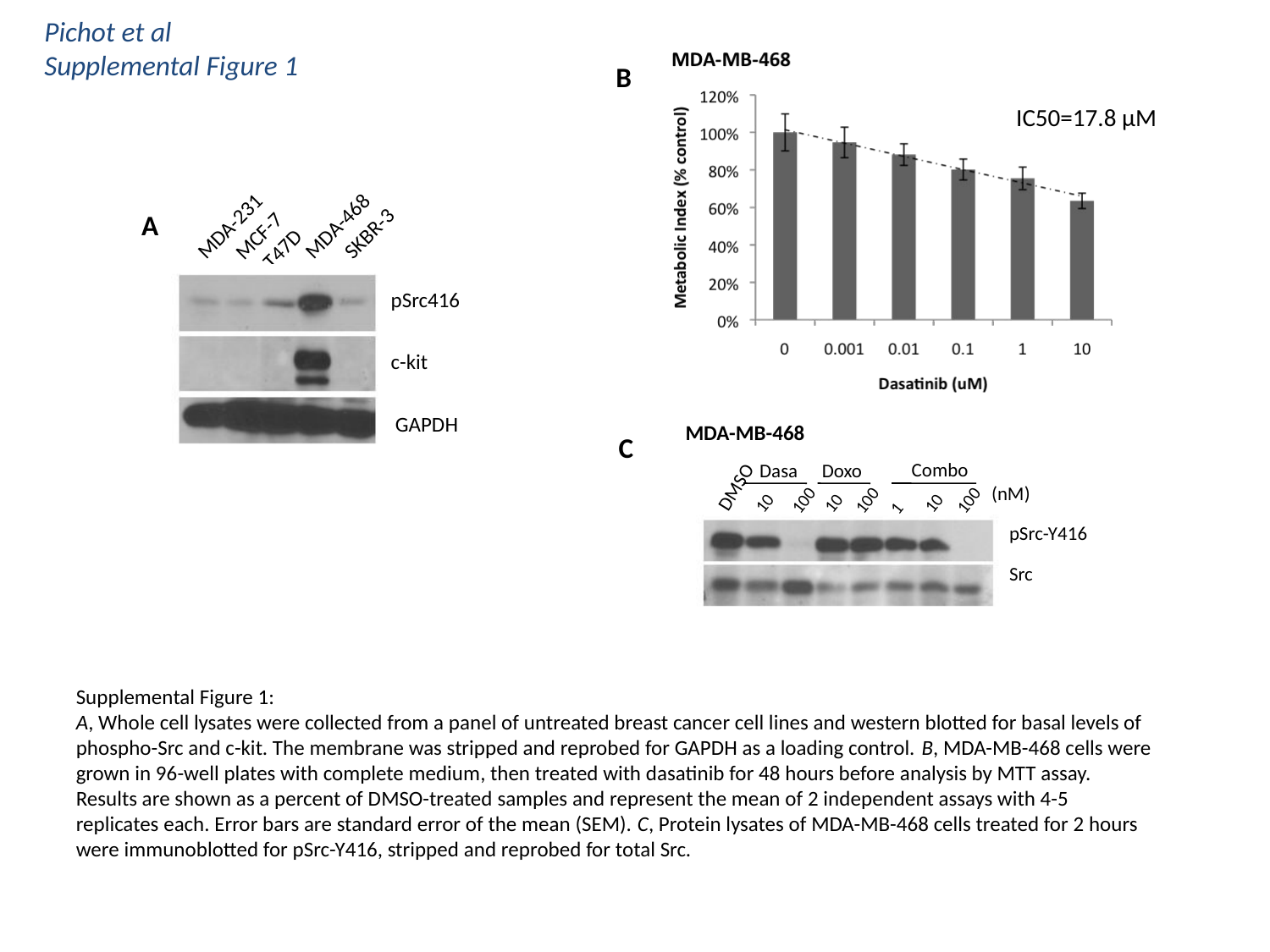

Pichot et al
Supplemental Figure 1
B
IC50=17.8 μM
MDA-231
MDA-468
SKBR-3
T47D
A
MCF-7
pSrc416
c-kit
GAPDH
MDA-MB-468
Dasa
Doxo
Combo
 (nM)
DMSO
100
100
100
10
10
10
1
pSrc-Y416
Src
C
Supplemental Figure 1:
A, Whole cell lysates were collected from a panel of untreated breast cancer cell lines and western blotted for basal levels of phospho-Src and c-kit. The membrane was stripped and reprobed for GAPDH as a loading control. B, MDA-MB-468 cells were grown in 96-well plates with complete medium, then treated with dasatinib for 48 hours before analysis by MTT assay. Results are shown as a percent of DMSO-treated samples and represent the mean of 2 independent assays with 4-5 replicates each. Error bars are standard error of the mean (SEM). C, Protein lysates of MDA-MB-468 cells treated for 2 hours were immunoblotted for pSrc-Y416, stripped and reprobed for total Src.
